# Compositional Characteristics of Glucuronide Conjugates in Regional *Glechoma hederacea* var. *longituba* Herbal Extracts Using a Set of Polyphenolic Marker Compounds

**DOI:** 10.3390/plants10112353

**Published:** 2021-10-30

**Authors:** Young Hye Hahm, Kun Cho, Yeong Hee Ahn

**Affiliations:** 1 Rophibio Inc., Osong-eup, Cheongju 28160, Korea; yhhahm@rophibio.com; 2 Center for Research Equipment, Korea Basic Science Institute, Cheongju 28119, Korea; chokun@kbsi.re.kr; 3 Department of Biomedical Science, Cheongju University, Cheongju 28160, Korea

**Keywords:** *Glechoma hederacea* var. *longituba*, polyphenol, glucuronide conjugate, compositional characteristics, LC–mass spectrometry

## Abstract

*Glechoma hederacea* var. *longituba* (GHL) is one of many herbal plants widely used in hot herbal teas and in oriental prescriptions to treat various diseases. Although the beneficial effects of GHL may be influenced by differences in the composition of active constituents in the herbal extracts, there are few reports on the compositional characteristics of GHL herbal extracts to date. In this study, liquid chromatography–mass spectrometry technology was used for comparative analysis of constituents in hot-water extracts of GHL samples obtained from various cultivating provinces in South Korea. A set of marker panel consisting of nine polyphenolic compounds, including glucuronide conjugates in particular, was constructed and used to monitor the compositional characteristics in each GHL extract. Our findings show that some of the marker compounds, including rosmarinic acid, were persistently observed as major constituents in the analyses of the 22 GHL sample extracts, whereas, interestingly, other marker compounds such as polyphenol-glucuronic acid conjugates displayed dramatic differences in compositional ratios. This chromatographic approach using the marker compound panel can be applied to qualitatively and quantitatively evaluate compositional characteristics in the GHL extracts, and can also be useful for quality assays of the GHL herbal plant in medicinal and industrial fields.

## 1. Introduction

Herbal plants have recently gained much attention in food and medicine due to their antioxidant phytochemicals, such as flavonoids and polyphenolic compounds [1,2,3,4]. Among such compounds, *Glechoma hederacea* var. *longituba* (GHL), commonly known as ground ivy or Keumheoncho in South Korea, has been distributed widely in Asia and Europe. GHL is often used in traditional oriental medicine as scientific evidence strongly shows that it exerts antioxidant, antibacterial, anticancer, anti-inflammatory, and immune-stimulating effects [5,6,7,8,9]. GHL has previously been used in treating asthma, cholelithiasis, urolithiasis, and various inflammatory diseases through ancient oriental prescriptions. GHL has been generally employed in a state of its total extracts or as a mixture together with extracts of other plants. Because the total extracts of GHL contain many constituents and its composition can have a major role in its biological effects, a compositional analysis of the chemical constituents in GHL extracts is necessary to not only verify the quality of the plant material, but also in utilizing the extracts in food and medicine.

The GHL extracts have been reported to contain various polyphenols [10,11,12,13], flavonoids [14,15], triterpenoids [16], and essential oils [17,18]. Among them, hydrophilic chemical constituents such as polyphenolic acids and flavonoids generally display physiochemical properties that allow for aqueous extraction and analysis using reverse-phase high-performance liquid chromatography (RP-HPLC). Because compositional characteristics of the chemical constituents in the total extracts of GHL may be important for evaluating the quality of the herbal plant in medicinal and industrial fields, a liquid chromatographic analysis for these major polyphenolic constituents can be alluring to evaluate the quality of the various GHL materials following various cultivating provinces, harvesting seasons, and processing conditions.

LC–MS methods provide high levels of sensitivity, allowing for in-depth compositional analysis and providing a full metabolic profile of plant samples [19]. Recently, we identified the major constituents of GHL hot-water extracts using high-performance liquid chromatography–high resolution mass spectrometry (HPLC–HRMS) and tandem mass spectrometry (HPLC–MS/MS) analysis [20]. The constituents identified from the extracts were hydrophilic small molecular polyphenols and polyphenol sugar conjugates rather than polyphenol aglycones. Furthermore, four of the major constituents were polyphenol glucuronide conjugates rather than polyphenol glycoside conjugates reported in previous analytical studies for GHL extracts [10,11,16].

In this study, a set of marker compounds consisting of major polyphenolic constituents in GHL extracts were established for practical chromatographic analysis. The marker compounds were then applied to qualitatively and quantitatively evaluate compositional characteristics among different GHL extracts obtained from various cultivating regions in South Korea.

## 2. Experimental

### 2.1. Materials and Reagents

Rosmarinic acid, formic acid, and caffeic acid were purchased from Sigma-Aldrich (St. Louis, MO, USA). Millex-HV syringe-driven filter unit was from Merk Millipore (Billerica, MA, USA). HPLC-grade water, methanol, and acetonitrile were purchased from TEDIA (Fairfield, OH, USA), and deionized water for HPLC analysis was purified using a Milli-Q system (Merck Millipore, MA, USA). Leucine encephalin for mass spectrometer tuning was obtained from Waters (Milford, MA, USA).

A total of 22 GHL plant materials were harvested from July to August in 10 different regions in South Korea. In 5 of the 10 regions, multiple samples were collected from different farmlands located in the region. The GHL plants were harvested in a state of whole plants including leaves and stems and dried in the shade at around 25–30 °C.

### 2.2. Preparation of the Hot-Water Extracts of GHL Sample

The harvested and dried GHL plants were cut into slices about 2~3 mm in length. Five grams of GHL slices were poured into 100 mL of distilled water. After 1 h incubation at 100 °C, the GHL hot-water extracts were cooled to 25 °C. Cellulose filters were used to remove plant material residue, and the volume of the extracts were adjusted. Each portion of the extracts was stored at −20 °C. Just before analyses, a portion of the prepared extracts was filtered by a Millex-HV syringe-driven filter (0.45 µm).

### 2.3. Total Phenol Content

Total phenolic content was analyzed using Folin–Ciocalteu reagent (sodium 3,4-dioxo-3,4-dihydronaphthalene-1-sulfonate) and by using gallic acid as a standard for the calibration curve. A total of 72 µL of distilled water and 80 µL of Folin–Ciocalteu reagent were added to 8 µL of extract solution. After 3 min, 8 µL of 10% sodium carbonate solution was added to the mixture, and the mixture was incubated for 1 h at room temperature with shaking in the dark. After incubation, the absorbance for the mixtures was measured in triplicate at 760 nm.

### 2.4. Radical Scavenging Activity

A total of 1,10-diphenyl-2-20-picrylhydrazyl (DPPH) methanol solution (0.2 mM) was prepared by mixing 25 mL of 0.4 mM methanol solution with 25 mL of Tris-HCl buffer (pH 7.4). We mixed 10 µL samples of GHL hot-water extracts with 90 µL methanol and 200 µL of the prepared DPPH methanol solution. After 1 h of incubation at room temperature in the dark, the absorbance of the mixture was measured in triplicate at 517 nm.

Butylated hydroxytoluene (BHT) was used as the positive control. Radical scavenging activity was calculated using the following equation: radical scavenging activity (%) = (absorbance of blank-absorbance of sample)/absorbance of blank × 100. The IC_50_ value was calculated by measuring the radical scavenging activity values obtained from 1:1 serial dilutions of each GHL extract.

### 2.5. HPLC Analysis

The extract (20 µL) was analyzed with an Agilent 1260 Infinity HPLC System equipped with a ZORBAX Eclipse Plus C18 (4.6 × 150mm, 3.5 um, Agilent, Palo Alto, CA, USA) and a UV detector. The mobile phase consisted of 0.2% formic acid solution (solvent A) and acetonitrile (solvent B). The flow rate was 1.0 mL/min, and a multilinear gradient was used: 0–1 min, 3–4% B; 1–90 min, 4–20% B; 90–94 min, 20–80% B; 94–99 min, 80–80% B; 99–100 min, 80–3% B; 100–110 min, 3–3%. The detection wavelengths were 330 nm. Each peak was identified on the basis of the comparison of retention time and mass value results obtained by LC–ESI tandem mass analysis. Heatmap visualization for the compositional characteristics of the marker compounds for the 22 GHL extracts was carried out using RStudio.

### 2.6. LC–ESI Tandem Mass Spectrometry

A high-resolution electrospray ionization (ESI) mass spectrometer was used to determine the structures of polyphenolic compounds. SYNAPT G2 mass spectrometer (Waters, Milford, MA, USA) coupled to a HPLC LC-20AD pump and SPD-20A UV–VIS detector (Shimadzu, Kyoto, Japan) was used for chromatographic and mass analysis. ZORBAX Eclipse Plus C18 column (4.6 x 150 mm, 3.5 um, Agilent, Palo Alto, CA, USA) was used to perform chromatographic separation at 25 °C using 1.0 mL/min flow rate and 5 µL sample injection volume. Mobile phase A (0.2% formic acid in water) and mobile phase B (100% acetonitrile) were applied to the following elution gradient program: 0–1 min, 3–6% B; 1–80 min, 6–20% B; 80–94 min, 20–80% B; 94–99 min, 80–80% B; 99–100 min, 80–3% B; 100–110 min, 3–3%. Electrospray ionization was run in a negative ion mode. The equipped analyzer was a hybrid tandem quadrupole IMS-TOF. SYNAPT G2 displayed 40,000 resolution (full width at half maximum). Scan speed was 20 spectra/s. Additional MS parameters included 100 °C source temperature, 300 °C desolvation temperature, 800 L/h desolvation gas flow, and 2000 V capillary voltage.

## 3. Results and Discussion

### 3.1. Total Phenol Content and Radical Scavenging Activity

**Figure 1:**
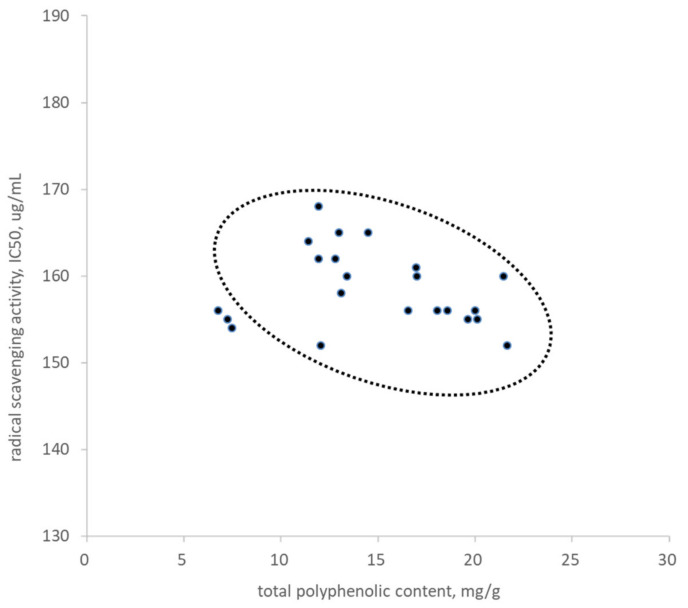
Total phenol content and scavenging effects of the hot-water extracts of 22 GHL extracts obtained from different provinces. Increasing total phenol content led to a proportional increase in radical scavenging activity, which was recoded with a decreased value of IC_50_.

Total phenol content for the 22 GHL extracts harvested from various growing regions was analyzed using the Folin–Ciocalteu method. The average phenol content for the 22 extracts was 14.81 ± 4.53 mg/g, as shown in Figure 1. Free radical scavenging activity was also analyzed for the 22 GHL extracts using the DPPH method. The average free radical scavenging activity (IC_50_) was 158.55 ± 4.45 mg/mL in the analytical condition. There was no significant regional tendency in the total phenol content and the free radical scavenging activity among the 22 different GHL extracts. These results were plotted against each other in Figure 1 to observe correlation. The results show that with increasing total phenol content, IC_50_ values tend to decrease, although some exceptions were also recorded. Previous studies have similarly shown that high phenol content is directly correlated to antioxidant activity in plant extracts [21,22].

### 3.2. Selection of Marker Compounds

Among the dried herbal plant samples obtained from several different provinces, a GHL sample (GS2; obtained from Goesan, Korea) was arbitrarily chosen for experimentation to obtain optimal HPLC analysis conditions [20]. Because GHL extracts consist of many constituents with varying abundance, we set up analysis conditions to concurrently differentiate the highest number of constituents within a single HPLC run.

**Figure 2:**
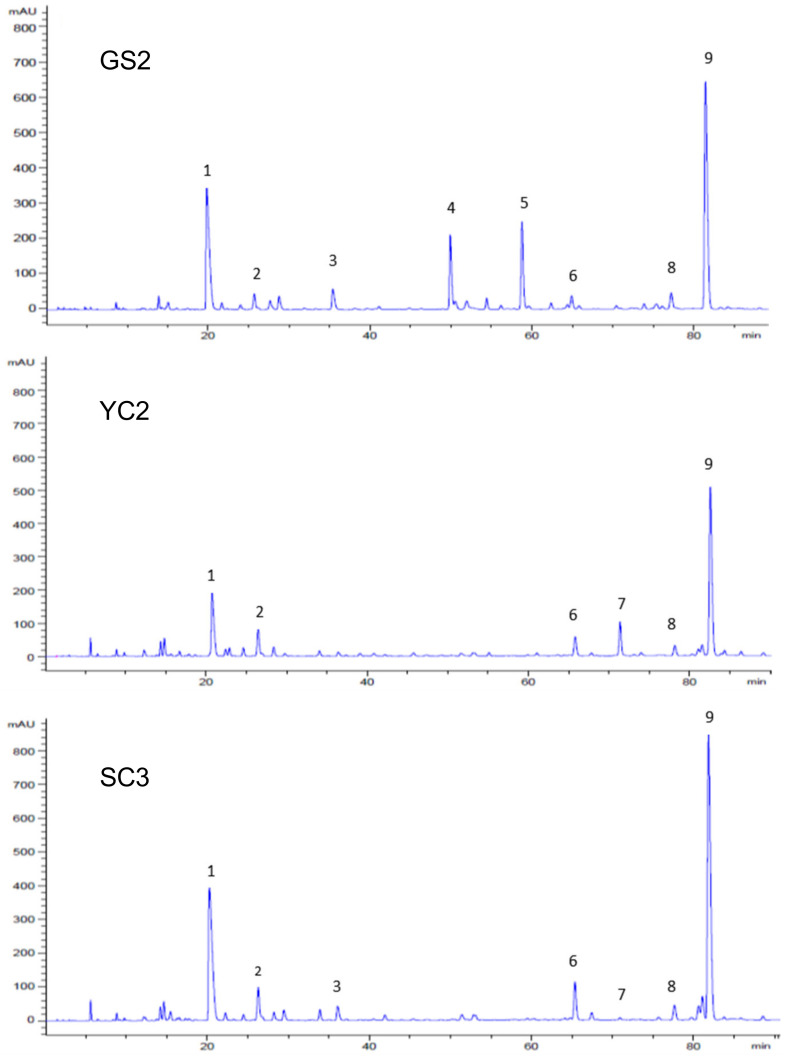
RP-HPLC chromatograms of three representative GHL extract samples.

The remaining 22 GHL extract samples were analyzed by RP-HPLC analysis, and three of the GHL extract samples exemplifying diverse peak patterns are displayed chromatographically in Figure 2. We discovered that under the same analytical conditions, the aqueous extracts of GS2 provided significantly different HPLC chromatogram patterns than that of YC2 (GHL sample obtained from Yeongcheon, Korea) and SC3 (GHL sample obtained from Sancheong, Korea). Many of the peaks appeared simultaneously in all three chromatograms with significant peak intensities. Among them, the outstanding peaks at 20 min and 82 min were recorded with very strong peak intensities. However, notable peaks at 50 min and 59 min for the GS2 sample were not recorded for YC2 and SC3 samples. Contrastingly, the peak recorded at 71 min for the YC2 sample was not recorded in the GS2 sample and was only observed as a minor peak for the SC3 sample. Following optimization of HPLC conditions, we selected major peaks showing significant peak intensities in the chromatograms as candidates of the marker compounds to evaluate compositional characteristics of the GHL extract samples.

As in Table 1, a set of marker compounds to evaluate regional difference among the GHL extracts was summarized. Among the selected markers, seven compounds were identified in our previously study [20]. Two marker compounds, glucopyranosyl rosmarinic acid (salviaflaside) and dihydroferulic acid 4-*O*-glucuronide, were newly identified by the high resolution ESI-MS and tandem mass spectrometry in the present study. Glucopyranosyl rosmarinic acid (marker compound 7, Rt = 71 min) was detected at *m/z* 521.1284 as [M-H]^-^ in the negative ion mode. The measured mass value displayed a mass accuracy of −2.1 ppm from the theoretical mass value *m/z* 521.1295 obtained from the expected molecular formula C_24_H_26_O_13_.

**Figure 3:**
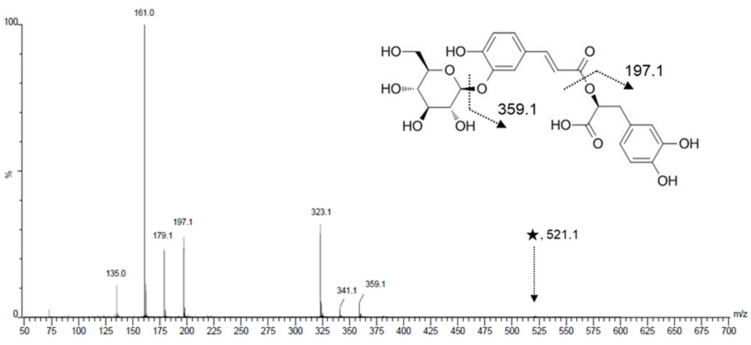
Tandem mass spectrum and fragmentation pattern of glucopyranosyl rosmarinic acid obtained by SYNAPT G2, HR–ESI–MS/MS. The symbol ★ represents the precursor ion, [M-H]^-^.

MS/MS spectrum of glucopyrnosyl rosmarinic acid obtained at the collision energy 30 V is illustrated in Figure 3. Fragmental ions generated at the precursor ion mass value, *m/z* 521.1, were detected at *m/z* 359.1, 341.1, 323.1, 197.1, 179.0, 161.0, and 135.0. Dihydroferulic acid 4-*O*-glucuronide (marker compound 3, Rt = 35.77 min) was also detected at *m/z* 371.0965 as [M-H]^-^ in the negative ion mode. The measured mass value displayed a mass accuracy of −3.4 ppm from the theoretical mass value *m/z* 371.0978 obtained from the expected molecular formula C_16_H_20_O_10_.

### 3.3. Quantitative Analysis of the Selected Markers Using HPLC

Since the object of this study is to develop a chromatographic method for evaluating and differentiating compositional characteristics among GHL extracts obtained from different cultivation sources, investigation of reproducibility and precision is imperative for practical usage of the current analytical method. Using marker compounds selected from major polyphenolic constituents in GHL extracts, we performed repeated analysis on extracts of GHL sample GS1 in order to investigate the reproducibility of the current HPLC analysis condition for evaluating compositional characteristics. Most marker compounds were quantitatively analyzed with good standard deviations (RSDs) using the optimized HPLC condition (Table 2). Although the range in the measured peak areas between the marker compounds are considerably large, all markers were analyzed with comparable RSDs, regardless of their relative abundances.

### 3.4. Compositional Characteristics between the Regional GHL Extracts

**Figure 4:**
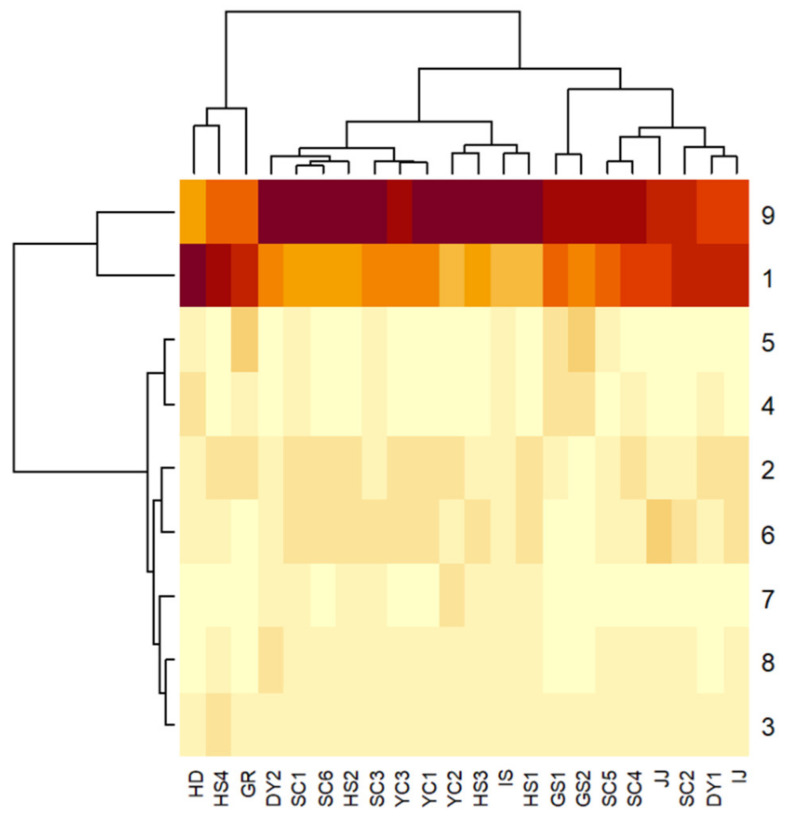
Heatmap visualization showing the compositional characteristics in each marker compound between the regional GHL extracts.

Next, the developed chromatographic method using the marker compounds was applied to the evaluation of compositional characteristics in each hot-water extract, prepared from GHL samples obtained from 22 different cultivating farmlands. Rosmarinic acid (marker compound 9) and caffeic acid (marker compound 1) were observed as the main compounds for all 22 GHL extracts, and their abundances are complementary with each other as shown in the heatmap visualization (Figure 4). Marker compound 1 showed superior abundance compared to marker compound 9 in GHL samples (HD, HS4, and GR). Some samples (JJ, SC2, DY1, and IJ) showed comparable abundance in marker compounds 1 and 2. However, other samples showed generally higher levels in marker compound 9 compared to marker compound 1.

**Figure 5:**
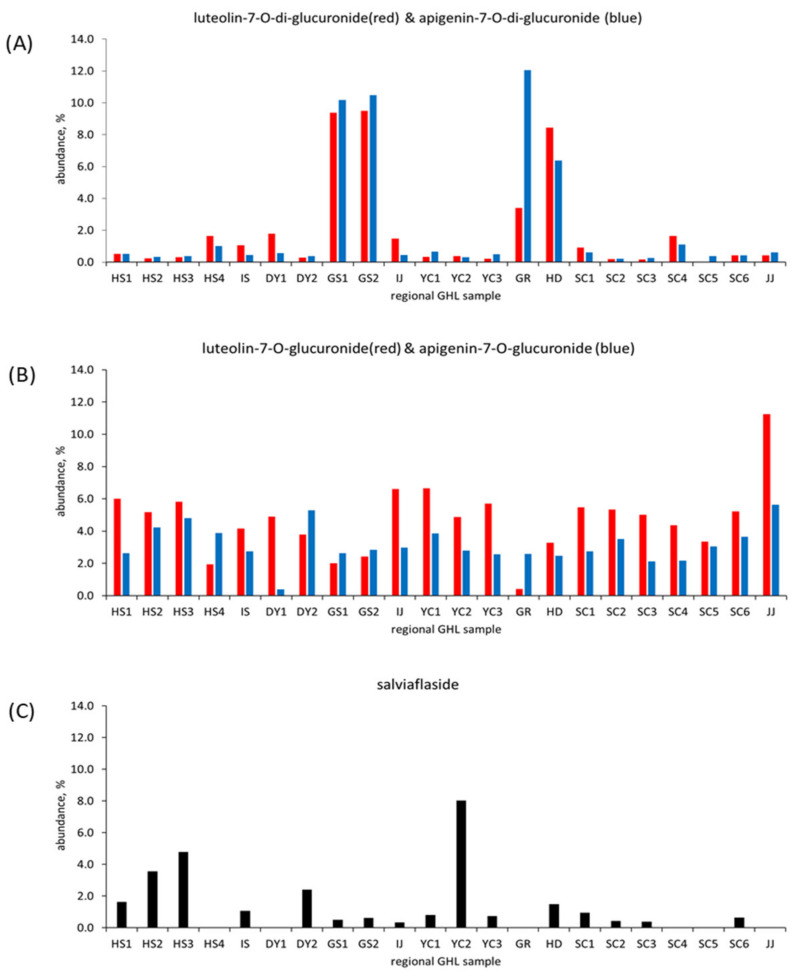
Comparison between the regional GHL extracts showing unique patterns in the compositional characteristics of (**A**) di-glucuronide conjugate, (**B**) mono-glucuronide conjugate, and (**C**) salviaflaside. Abundance represents the ratio of the peak area of a marker to the overall peak area recorded on the HPLC chromatogram of each GHL extract.

Interestingly, it should be noticeable that some marker compounds showed significant characteristics in minor polyphenolic compositions based on geographical distribution, as illustrated in detail in Figure 5. In Figure 5A, we observed that di-glucuronide derivatives such as luteolin-7-*O*-di-glucuronide and apigenein-7-*O*-di-glucuronide showed significant dual peak intensities for GHL extracts obtained from Goesan, Gurye, and Hadong provinces. These results indicate their significance as marker compounds. Contrary to di-glucuronides, mono-glucuronide derivatives such as luteolin-7-*O*-glucuronide and apigenin-7-*O*-glucuronide showed similar peak intensities for all GHL extracts, as shown in Figure 5B. In this study the polyphenol glucuronide conjugates were identified with significant abundance, rather than their polyphenol aglycones or glycoside conjugates often reported in previous studies [11,23].

These glucuronide conjugates of flavonoids are produced biosynthetically from the reaction of UDP-glucuronate and flavonoid by glucuronosyltransferase enzyme [24]. Microbial glucuronidation of polyphenols was also attempted in order to obtain potential therapeutic agents [25]. The glucuronidation of polyphenol substances can affect their bioavailability through absorption in enterocytes and the biological activity of the dietary polyphenol substances [26]. Since a large number of in vitro studies are often conducted on the basis of the dosages of polyphenol aglycones rather than their active metabolites such as the polyphenol glucuronides, abundant care is required for accurate evaluation of the beneficial effects of dietary polyphenols regarding which type of polyphenols play practical roles in physiological processes.

On the other hand, glucopyranosyl rosmarinic acid (marker compound 7, salviaflaside) showed significantly varying peak intensities for the analyzed regional GHL extracts, as displayed in Figure 5C. Salviaflaside was identified as one of the major polyphenol compounds from the extracts of *Prunella vulgaris* L. spica [27] and chia seeds [28]. Salviaflaside displayed notably high abundance in the analyses of the GHL extracts prepared from HS17, HS18, DY19, and YC18 samples, which is significantly different from the patterns observed for mono- and di-glucuronide derivatives for the same extracts. These results also indicate the significance of salviaflaside as a marker compound along with di-glucoronide derivatives. The compositional characteristics for each of the regional GHL extracts may be influenced from a variety of cultivation conditions, such as soil, climate, and harvesting methods. With the results obtained in the present study, it is difficult to fully understand the main cause for the variations in composition among the GHL extracts. However, we were able to practically investigate the compositional characteristics among many regional GHL samples by using the marker compound panel.

## 4. Conclusions

Liquid chromatography has been widely used for evaluation of compositional features in various extracts as well as identification of constituents of interest in the field of herbal botanicals. In the present study, HPLC technology was applied to evaluate compositional characteristics of hot-water extracts for GHL samples obtained from different growing provinces in South Korea. For our analysis, we first constructed a set of marker compounds to compare compositional characteristics among the GHL extracts. The marker compound panel consists of constituents persistently showing high abundance and/or uniquely detected in a few samples. The constructed marker panel facilitated our comparative analysis for each chromatogram by clearly differentiating compositional characteristics among the GHL samples. Because the GHL herbal plant is usually traded as dried materials in the herbal commercial field, its identification and quality evaluation may be not satisfactory solely by appearance. Therefore, the simple and practical chromatographic technology presented in our study using marker compounds can be useful for clearly evaluating quality of GHL materials in herbal inspection and in industrial fields. Furthermore, it can be useful in examining compositional features for herbal raw materials by varying growing provinces and cultivation conditions.

## Figures and Tables

**Table 1:** Marker compounds selected from the analyses of the GHL extracts.

Peak No.	Identified Compound	Rt (min)	Formula	Theo. Mono-Isotopic Mass	Theo. [M-H]^−^ (*m/z*)	Exp. [M-H]^−^ (*m/z*)	Δ, ppm
1	{[(2E)-3-(3,4-Dihydroxyphenyl)-2-propenoyl]oxy}malonic acid	20.15	C_12_H_10_O_8_	282.0376	281.0298	281.0294	−1.3
2	Trans-caffeic acid	25.99	C_9_H_8_O_4_	180.0423	179.0345	179.0341	−2.1
3	Dihydroferulic acid 4-*O*-glucuronide	35.77	C_16_H_20_O_10_	372.1056	371.0978	371.0965	−3.4
4	Luteolin-7-*O*-di-glucuronide	50.35	C_27_H_26_O_18_	638.1119	637.1041	637.1043	0.4
5	Apigenin-7-*O*-di-glucuronide	59.16	C_27_H_26_O_17_	622.117	621.1092	621.1091	−0.1
6	Luteolin-7-*O*-glucuronide	65.24	C_21_H_18_O_12_	462.0798	461.0720	461.0711	−1.9
7	Glucopyranosyl rosemarinic acid, salviaflaside	70.69	C_24_H_26_O_13_	522.1373	521.1295	521.1284	−2.1
8	Apigenin-7-*O*-glucuronide	77.48	C_21_H_18_O_11_	446.0849	445.0771	445.0765	−1.3
9	Rosmarinic acid	81.72	C_18_H_16_O_8_	360.0845	359.0767	359.0765	−0.5

**Table 2:** Triplicate analysis of GS1 using the optimized HPLC analysis condition.

Marker Compound	Area (mAU * s)			Av. Area (mAU * s)	STDEV	RSD, %
	Run 1	Run 2	Run 3			
1	669.1	667.5	627.7	654.8	23.4	3.6
2	130.5	135.2	127.0	130.9	4.1	3.2
3	89.6	92.6	87.0	89.7	2.8	3.1
4	242.9	250.6	237.4	243.6	6.6	2.7
5	263.6	271.7	257.7	264.3	7.0	2.7
6	56.1	57.9	54.4	56.2	1.8	3.2
7*	n.d.	n.d.	n.d.	n.d.	n.d.	n.d.
8	61.5	63.8	59.9	61.7	1.9	3.1
9	843.4	873.5	820.3	845.7	26.6	3.2

* Marker compound 7 was not detected in the analysis of the extracts, GS1.
